# Role of Zinc Supplementation in the Improvement of Acute Respiratory Infections among Iranian Children: A Systematic Review

**Published:** 2020-01

**Authors:** Mozhgan Moshtagh, Rana Amiri

**Affiliations:** 1 Social Determinants of Health Research Center, Faculty of Health, Birjand University of Medical Sciences, Birjand,Iran,; 2 Visiting Scholar, Department of Geography and Environmental Sciences, Northumbria University, UK.

**Keywords:** Zinc supplementary, Respiratory infection, Children, Iran, Systematic review

## Abstract

**Background:**

Respiratory infectious disease is considered as one of the most serious problems among children in developing countries. The importance of zinc in the improvement of respiratory disease has been indicated. However, there are some unanswered questions and uncertainty. This systematic review aimed at assessing the therapeutic benefits of zinc supplementation on respiratory infections of Iranian children.

**Materials and Methods:**

Databases, such as PubMed, Scopus, Embase, Magiran, and IranDoc, were searched for randomized controlled trials published from January 1998 to December 2017 on Zinc supplementation for the treatment or improvement of acute respiratory disease among Iranian children, during March 2018. Studies were screened according to the PICO framework, and irrelevant studies were excluded.

**Results:**

A total of 5 studies conducted on 522 children were included in this review. Except for one study, others had indicated the beneficial effect of zinc supplement on improving signs and symptoms of respiratory infectious disease and earlier discharge from the hospital.

**Conclusion:**

Although studies on the efficacy of zinc on respiratory infectious disease of children in Iran have not widely considered and more studies should be conducted, all published articles (except for one of them) had indicated the effectiveness of zinc supplementation on respiratory infections among children. Other criteria, such as age, gender, birth weight, diet, and type of respiratory infections, should be considered during zinc therapy. Controlling these confounding variables and measuring the level of blood zinc are necessary to understand how much zinc should be prescribed for respiratory treatment of children.

## INTRODUCTION

Acute respiratory infections, such as pneumonia, are the most common underlying causes of illness and mortality among children (1–4). Their prevalence of mortality is about 2 million deaths per year among children in low-income countries (5, 6). Malnutrition, low birth weight, high population density, and air pollution are some most important risk factors for pneumonia in developing countries (7, 8). Climate changes, rising air pollution, and population density in major cities of Iran in recent years have led to the further spread of such infections among vulnerable populations, such as children. Respiratory infections may progress gradually to chronic infections that are difficult to treat. Therefore, interventions with positive effects on the treatment process and reduction of respiratory complications in children are valuable (9).

Zinc is one of the most important micronutrients, and its deficiency is common in developing countries, such as Iran. Malnutrition, lower level of meat consumption, and a higher level of consumption of cereals, such as white rice containing less zinc are some causes of zinc deficiency among children living in developing countries (1, 10). Zinc deficiency in different cities of Iran has been reported in different cities of Iran (11–14). Low levels of zinc in the blood of children can increase the risk of respiratory infections (1, 15). Due to the protective and anti-inflammatory properties of zinc, it plays an important role in preventing respiratory infections among children and promotes their health (7, 16, 17).

Several studies on various populations and age groups have indicated the role of zinc supplementation in improving the immune system and preventing infectious diseases (5, 18, 19). However, due to the contradictory evidence (20–22), and the effect of some variables, such as socioeconomic status, geographical location, race, ethnicity, age, and gender on the level of zinc (23–25), these confounding variables should be considered and controlled for an accurate evaluation. Some studies have been conducted in Iran on the effect of zinc on the prevention or treatment of infections (26, 27, 28, 29, 30, 31). However, some questions should be answered regarding the effectiveness of zinc on clinical outcomes and its impact on health policymaking. For example, the following questions should be addressed: Is zinc supplement associated with a positive effect on respiratory disease in children in studies conducted in Iran? What criteria has considered as a positive outcome or efficacy of an intervention? Does zinc supplementation affect respiratory infections in Iranian children and what criteria in these studies should be considered? Can zinc supplements be useful in reducing the economic costs of respiratory infections among Iranian children? What is the effect of zinc supplementation in different groups (age, gender, weight, and type of infection or treatment)? Which criteria and methods are the most effective or associated with the most favorable results?

Considering the high incidence of respiratory infections in Iran and the high prevalence of zinc deficiency among children, as well as to reduce the burden of disease (complications or mortality), and promote health and well-being in the Iranian society, this study aimed at evaluating the effectiveness of zinc supplementation to improve respiratory infections (symptoms and outcomes) among Iranian children.

## MATERIALS AND METHODS

This review was performed based on the guideline for meta-analyses and systematic reviews (PRISMA). To carry out this research, researchers initially reviewed studies conducted in Iran. The framework and strategy for screening studies were in line with the research question and the patient intervention comparator outcome (PICO) process. To evaluate the effectiveness of zinc supplementation on respiratory infections in Iranian children, randomized controlled trials (RCTs) assessing the effect of zinc supplementation on the improvement of respiratory infections in Iranian children were selected. Inclusion criteria were using eligible subjects, studies matched with the aims of the current study in terms of method, intervention, subjects, and outcome.

### Search strategy

Databases, such as PubMed, Scopus, and Embase were searched in March 2018 for RCTs published from January 1998 to December 2017 using the following keywords: “Zinc” AND “Iranian children,” “Zinc” AND “Iran” AND “children,” “Zinc insufficiency,” “Zinc deficiency,” “Zinc status,” “Zinc” AND “Respiratory infection,” “Zinc” AND “Pneumonia” with the words “children” and “Iran,” also in combination with “Iranian children.” Iranian databases, such as Magiran and IranDoc, were also searched using identical Persian keywords. For manual search, references of the retrieved articles were reviewed.

### Inclusion criteria

The inclusion criteria were RCTs conducted on children less than 6 years old or preschoolers at hospitals or clinics in different parts of Iran aimed at studying the effect of zinc supplementation (syrup) on the respiratory infection. Therefore, studies conducted on non-target age groups and in other countries (except Iran), as well as descriptive-analytical studies and interventional studies with no relevant outcomes based on the targeted dependent variable (respiratory infections) were excluded.

### Exclusion criteria

Studies conducted in other countries, research on adolescents, adults, and elderly populations, studies that examined other micro-nutrients individually or in combination, research on the effects of various diseases on zinc deficiency in children, clinical trials that measured the effect of zinc on other disorders, diseases, and infections were excluded. Also, non-interventional studies that investigated the effect of zinc supplementation on the prevention of infection in outpatient and non-hospitalization cases were removed.

## RESULTS

During the search process, 2150 studies were found. After three stages of screening based on the studies’ titles and abstracts, 7 studies were eligible for full-text appraisals. After reviewing their full-text, one of the trials (4 groups, including three intervention groups and one control group) that was performed on children who referred to the healthcare center and evaluated the effect of zinc on the prevention of respiratory infections was deleted. Accordingly, only six studies with similar characteristics (RCTs with a control group) were selected for data analysis.

### Description of the studies

We could not access to the full-text of one study evaluated the effect of routine zinc supplementation on pneumonia in children aged six months to 3 years old (New Medical Journal, No. 161, June 26, 2002). Of five available studies (one in Persian and four in English), a total of 522 children with respiratory infections admitted to hospitals were assessed. Some characteristics of the participants are shown in Table 1.

**Table 1.** Participants’ characteristics in the studies

**Study**	**Age (month)** Mean (SD)		**Mean Weight (Kg)** Mean (SD)		**Gender (percent)**	

	**Case**	**Control**	**Case**	**Control**	**Case**	**Control**
Heydarian et al. (30)	5.6 ± 3.62	5.64 ± 3.01	?	?	M: 72	M: 60
					F: 28	F: 40
Valavi et al. (27)	15.41	15.89	10.13	10.19	M: 54.1	M: 51.6
					F: 45.9	F: 48.4
Habibian et al.(31)	20.97±21/2	21.13±24	10.3±4.8	10.1±4.7	M: 62.9	M: 56.5
					F: 37.1	F:43.5
Qasemzadeh et al.(28)	<6: 38.34%	<6: 41.67%				
	6–24: 53.34%	24–60: .34%	?	?	M: 58.3	M: 56.7
	6–24: 50%	24–60: .34%			F: 41. 7	F: 43.3
Mahyar et al.(29)	7 ± 10	7 ± 13.6	8.1±2.1	7.3±2	M: 52	M: 58 M
	Med(IQR)	Med(IQR)			F: 48	F: 42 F

### Age

Studies done by Valavi et al. (2011) and Qasemzadeh et al. (2014) were quite similar according to the age group and number of participants (27, 28). According to Valavi et al. study (27), 43.9% of the participants were less than one year old. In Qasemzadeh et al. study (28), gender and age were considered and controlled during the study. There were three age groups of less than 6 months, 6 months to 1 year, and 1 to 5 years. Mahyar et al. (29) and Heydarian et al. (30) had assessed children aged less than two years (2 to 23 months and 2–24 months), and Habibian et al.(31) had an extensive age range of subjects.

### Gender

In all studies, the proportion of male to female in both control and intervention groups was almost equal. Except for Heydarian et al. (30) study, this proportion was 74 boys and 50 girls.

### The number of subjects

Regarding sample size, there was a fairly large difference between the studies. The highest and lowest sample sizes were 128 and 50 children, respectively.

### Demographic criteria or disease diagnosis

Regarding differential diagnosis of the disease, in four studies, subjects had been selected based on two specific diagnoses (pneumonia (27, 28) and bronchitis (29, 30)). In one study, children with different kinds of respiratory infections were entered. The highest percentage (29%) was attributed to bronchitis, and other diseases, such as viral pneumonia, bacterial pneumonia, and the common cold, respectively (31). The onset of symptoms before the study was almost less than one week.

### Methodologies of studies

Except for one study (28), other studies reported inclusion and exclusion criteria. In the study done by Heydarian et al. (30), the random allocation method of the subjects into two groups and the type of blindness was unclear; however, they had described how they fed the participants (2–23 months) in detail. In the intervention group, 20 infants had been fed with breast milk and five others with formula milk or other foods plus breast milk. In the control group, 17 infants had been breastfed, and eight others had used a combination of breast milk and formula milk. According to Mahyar et al. (29) study, children had acute bronchitis and, for the first time, experienced respiratory whizzing and difficulty (based on parental report and pulmonary radiography).

Demographic characteristics of the subjects (age, weight, height, and head circumference), kind of feeding (breast milk or other items), exposure to cigarettes at home, hemoglobin level, and clinical and paraclinical measures (gasometry) were the important and positive features of the Mahyar et al. study in the methodology section. Furthermore, the blindness of the mothers, care providers, researchers, and evaluators of group allocations (assignment to groups using random numbers on the zinc sulfate bottle) and providing details about zinc supplementation (manufacturer, the standard code, and the standard dose) were other strengthens of the studies (29).

On the other hand, there were some ambiguities in the methods of the study. It was stated that the two groups were matched regarding confounding and underlying variables (age, height, weight, gender, head circumference, type of nutrition, etc.); however, in other parts of the study, it was noted that the participants were randomly divided into groups.

In Qasemzadeh et al. study, limited criteria had been used for examining the clinical symptoms before the intervention (28). In Valavi et al. (27) study, besides reporting the inclusion and exclusion criteria, differential diagnostic criteria (severe pneumonia) in both age groups were found. Also, given that diarrhea could disrupt zinc absorption, considering this variable as an exclusive criterion had a great impact on the accuracy of the study. Also, reported power, calculated error, and blindness of the researchers, care providers, and subjects about the type of intervention were other features of this study.

**Duration and type of treatment:** In Habibian et al. study, varied age groups and clinical diagnosis had been used; however, limited evidence had been provided about the kind of intervention for each disease and comparison between them (31). In Heydarian et al. study, although the name of the medicine had been reported based on the diagnosis of the disease in each group, no data were found about the symptoms of disease and outcome of the treatment before and after the intervention (30). The amount of zinc in Mahyar et al. study was for five days with a fixed dose of 5 mg per 12 hours (10 mg/day) for children aged less than one year, and 20 mg/day (10 mg every 12 hours) for children aged over two years old (29).

Qasemzadeh et al. had used 5 ml per 12 hours zinc supplementation plus antibiotic (the name of the antibiotic was not mentioned) for the treatment of the subjects (28). In Valavi et al. (27) study, the protocol of treatment and the amount of zinc supplementation (2 mg zinc per kg body weight, once daily) plus ampicillin or cefazolin (intravenous) depending on the type of bacteria had been determined clearly. In case of no response after 48 hours, ceftriaxone or vancomycin had been replaced and continued during hospitalization, and vital signs had been assessed every 8 hours. The ambiguity in this study was a contradiction in reporting the duration of treatment by zinc supplements. The duration of receiving zinc supplement in the abstract of the article was five days, whereas, in the methods section, it had been mentioned during the hospitalization period.

Almost all studies had reported some positive changes in vital signs and clinical symptoms. The duration of admission and the length of treatment had been reported as positive signs for zinc therapy. Habibian et al. had reported a decrease in fever episode, coughing, and hospitalization period in the intervention groups compared with the control group (31). In Heydarian et al. (30) study, improvements in the symptoms (cyanosis, whizzing, and tachypnea) and response to treatment had been observed after 24 hours in the control group. The result of the study showed that zinc supplementation was ineffective for improving the symptoms in the intervention group. In this study, the number of male infants in the intervention group was more than twice the number of females. The result of this study was different from other studies due to the high intra-group variations and the effect of gender on the intervention group. Also, according to the report, on the second and third days of the treatment, the two groups did not show a significant difference regarding respiratory distress.

In Mahyar et al. study, clinical symptoms of the patients based on the researcher’s criteria had been presented in both groups before and after the treatment. During the first 72 hours of the treatment, 49 out of 50 patients in the intervention group showed symptoms’ relief and had a shorter hospitalization period, but only 26 patients in the control group had this characteristic (29).

The outcomes of Valavi et al. study had been precisely reported based on the time of the first stage (improvement of the symptoms, such as fever, respiratory distress, and improvement of all signs of the disease), and the second stage (hospitalization period) (27). The duration of follow-up after the intervention had not been accurately mentioned in Qasemzadeh et al. (28) study. In this study, treatment outcomes (clinical signs, chest x-ray, and hospitalization period) had been found in two groups. The general characteristics of the studies are presented in Table 2, and some important criteria regarding the quality assessment of the studies are provided in Table 3.

**Figure 1.**
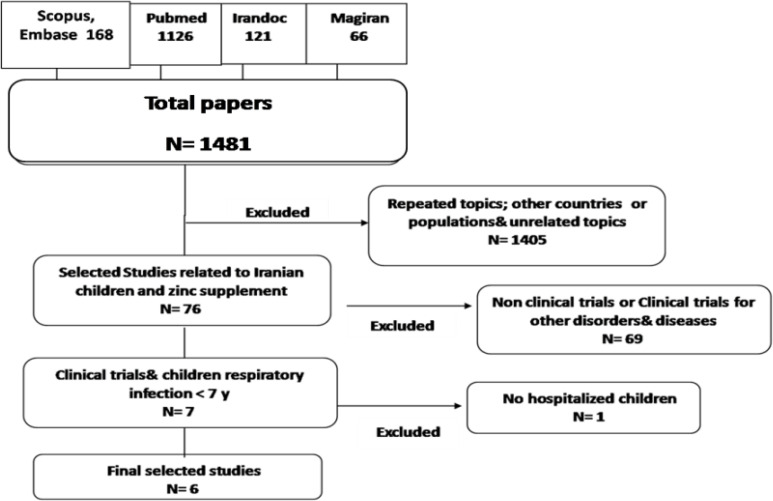
Screening and selection of studies

**Table 2.** Comparison of the general characteristics of the studies

**Study**	**Trial**	**Follow-up (days& h)**	**Disease**	**Treatment (zinc sulfate)**	**Participants (N)** Age (month)	**Outcomes**
Heydarian et al.(30)	RCT (D-blind)	Admission 24, 48 & 72h	Bronchiolitis	1cc/kg < 1 y 10cc > 1y	N: 50 2 – 23	RSS in control group at first day (P=0.04), but no differences At days 2 and 3 (P=0.9)
Valavi et al.(27)	RCT (blind)	Primary: RSS Secondary: LoS	Severe pneumonia	2 mg/kg/d	N: 128 3 – 60	RSS (P<0.001)&LoS (p<0.001)
Habibian et al.(31)	RCT	5 d	Pneumonia, Bronchiolitis, Common cold	5mg/12 h	N: 124 1–108	RSS (p<0.001)& LoS (p=0.42)
Qasemzadeh et al.(28)	RCT (D-blind)	< 2 d 2–5 d > 5d	Pneumonia	5mL/12 h	N: 120 3–60	RSS (P= 0.044) & LoS (P = 0.004).
Mahyar et al.(29)	RCT or matching (blind)	24, 48, 72, 96 & 120 h (5days)	Bronchiolitis	5mg/12h < 1 y (10mg/d) 10 mg/12h > 1y (20mg/d)	N: 100 2 – 24	RSS (48–72 h), (P=0.0001) LoS (p<0.023)

RSS: Relieve signs& symptoms, LoS: Length of Stay (hospitalization time), D-blind: double-blind, d: days, h: hours

**Table 3.** Methodological quality of included studies

**Study**	**Randomization**	**Allocation Concealed**	**Baseline similarity of groups**	**Specified eligibility criteria**	**Blinded care provider**	**Blinded patient**	**Intentional Analysis**
**Heydarian et al.(30)**	**+**	**+**	**+**	**+**	**¿**	**+**	**+**
**Valavi et al.(27)**	**+**	**+**	**+**	**+**	**+**	**+**	**¿**
**Habibian et al.(31)**	**+**	**¿**	**+**	**+**	**¿**	**¿**	**¿**
**Qasemzadeh et al.(28)**	**+**	**+**	**+**	**−**	**+**	**+**	**¿**
**Mahyar et al.(29)**	**¿**	**+**	**+**	**+**	**+**	**+**	**¿**

## DISCUSSION

None of the studies had measured the level of zinc in the blood of children before or after the intervention. According to the studies, the child’s birth weight, geographical area, air pollution, and population density are effective on the level of zinc in plasma (7, 8). Therefore, measuring blood zinc levels in children before and after the intervention is useful to determine the effectiveness of the treatment (26, 32).

The contradictory findings in the previous studies can raise questions for the subsequent studies. For example, Heydarian et al., in their study, found that zinc supplementation is not effective on respiratory infections, whereas other studies found opposite findings and showed that zinc could improve clinical symptoms. Therefore, it can be asked whether this contradiction can be linked to zinc deficiency in their blood or whether the level of zinc blood in Heydarian et al. study was normal and in other studies was lower than normal. Also, the type of response to treatment among children with different levels of zinc in their blood can be considered (30). Further prospective studies (cohorts) or clinical trials with larger sample sizes comparing children with different zinc serum levels should be conducted.

Furthermore, examining the differences in clinical and paraclinical symptoms before and after the intervention will address the ambiguities. Mahyar et al.(29), in their study, have discussed whether their result is positive compared with Heydarian et al.(30) study is because of prescribing a constant and greater dose of zinc or according to the weight of a child (10 mg) and a greater sample size. Given the fact that the participants in the two studies were similar in terms of age and clinical diagnosis, such a justification seemed logical. Although precise evidence, such as level of zinc in the blood before and after the intervention, clinical tests (measuring complete blood count (CBC) and serum Iron), and paraclinical evaluations (determining the type of virus) may help more to clarify the different results among studies.

As some studies used zinc supplementation based on the weight of the child, and others applied an equal dose for all participants with different weight values, subjects with a lower weight and at lower ages should be more benefited. According to this hypothesis, considering that the mean dose of zinc supplement in Vavali et al.(27) study was higher than other studies (2 mg/kg), and in Qasemzadeh et al.(28) study, an equal dose for different ages (3–60 months) was used, therefore, children less than one-year-old might have received higher levels of zinc. If these studies had examined the effect of zinc between groups (case and control) and inter-group pattern, they could partly answer to this hypothesis. In Valvali et al.(27) study, severe pneumonia had been diagnosed by the number of the respiratory rate per minute, and according to the definition of the variables in the study, this scale varied among children aged 3–12 months or older. Therefore, reporting this criterion based on the age group and time (before and after intervention) could have provided a more accurate assessment of the outcomes of the study. In addition, the number of leukocytes had been reported only before the intervention. Comparing this criterion before and after receiving zinc supplementation could express its effectiveness and role more clearly. Reducing the fever episodes in the findings of this study and other studies may be related to the reduction of cytokines and inflammatory reactions in the intervention groups (33).

In Mahyar et al. study (29), the demographic characteristics of the subjects regarding age, weight, height, head circumference, exposure to cigarettes at home, hemoglobin levels, and clinical and paraclinical parameters (arterial blood gas) had been reported. Providing these variables in Heydarian et al. study (30), could present a more accurate assessment of the consequences. Although the main medicine had not been mentioned in Mahyar et al. study (29), the use of bronchodilator and zinc supplements may indicate the viral bronchitis of the subjects. In Heydarian et al. study (30), the name of the main medicine had not been mentioned, as well. Clear reporting of main medicine and clinical diagnosis of the subjects in both studies could help for a better assessment of outcomes.

In Habibian et al. (31) study, varied age groups, and differences in the diagnosis of the disease in participants were observed. A detailed report regarding the treatment process in different age and gender groups could partly clarify the effectiveness of outcomes.

Due to the contradictory results of the included studies, the following questions can be raised: 1) is this difference due to the different doses of zinc supplementation or the patient’s characteristics (age or gender), stage of the disease, type of treatment (prescription of an antibiotic and its type), viral or bacterial infections, or zinc plasma levels before the intervention?; 2) What is the therapeutic mechanism of zinc supplementation in different viral and bacterial infections?; and 3) Is the response to zinc supplementation associated with the type, dose, and duration of antibiotic treatment? Microbiological tests to determine the type of virus and bacteria, as well as response to the treatment are helpful to clarify these questions.

Also, despite the importance of child feeding, the role of this variable had not been adequately addressed in included studies; only in three studies (Valavi et al. Mahyar et al., and Heydarian et al.), children who were breastfed or were formula-fed had been mentioned (27,29, 30). Due to the important role of vitamins A and D on inflammation, daily intake of these vitamins in children should be considered.

## LIMITATIONS

In this review, studies conducted in Iran were reviewed, and the results cannot be generalized. One of the most important limitations of our review was the lack of access to unpublished reports. Due to the lack of standardization of some studies or reporting findings inaccurately (before and after the intervention), combining the results, comparing the effect size, and analysis, the findings of the studies were impossible.
